# *Elaeagnus umbellata*: A miraculous shrub with potent health-promoting benefits from Northwest Himalaya

**DOI:** 10.1016/j.sjbs.2023.103662

**Published:** 2023-05-02

**Authors:** Mujtaba Aamir Bhat, Awdhesh Kumar Mishra, Mohammad Azhar Kamal, Safikur Rahman, Arif Tasleem Jan

**Affiliations:** a Gene Expression Lab, Department of Botany, Baba Ghulam Shah Badshah University, Rajouri 185234, India; b Department of Biotechnology, Yeungnam University, Gyeongsan 38541, Republic of Korea; c Department of Pharmaceutics, College of Pharmacy, Prince Sattam Bin Abdulaziz University, Alkharj 11942, Saudi Arabia; d Department of Botany, Munshi Singh College, BR Ambedkar Bihar University, Muzaffarpur 845401, Bihar, India

**Keywords:** Antioxidant, Disease prevention, *Elaeagnus umbellata*, Phytochemicals, Therapeutics

## Abstract

Medicinal plants encompassing a series of bioactive compounds have gained significant importance for use in the treatment of different diseases. Of them, *Elaeagnus umbellata* Thunb. (Deciduous shrub found in dappled shade, and sunny hedge) exhibits high medicinal value, with a widespread distribution across the Pir Panjal region of the Himalayas. Fruits serve as an excellent source of vitamins, minerals, and other essential compounds that exhibits hypolipidemic, hepatoprotective, and nephroprotective effects. The phytochemical fingerprint of berries revealed them to have a high content of polyphenols (with major proportion of anthocyanins), followed by monoterpenes and vitamin C. Extract of fruits help in regulating the digestion and absorption of glucose and reduces inflammation and oxidative stress. The phytosterols upholding anticoagulant activity serve the purpose of causing decrease in angina and the blood cholesterol levels. Phytochemicals such as eugenol, palmitic acid, and methyl palmitate exhibit potent antibacterial activity against broad range of disease-causing agents. Additionally, a high percentage of essential oils attribute it with the property of being effective against heart ailments. The present study highlights the importance of *E. umbellata* in traditional medicinal practices, and summarizes the knowledge of its bioactive constituents and a snapshot vision of remarkable biological activities like antimicrobial, antidiabetic, antioxidant, etc towards understanding its role in the development of efficient drug regimens for use in the treatment of different diseases. It also underlines the need to explore the plant on nutritional aspects to strengthen the existing knowledge pertaining to health promoting potential of *E. umbellata*.

## Graphical abstract

**Figure:**
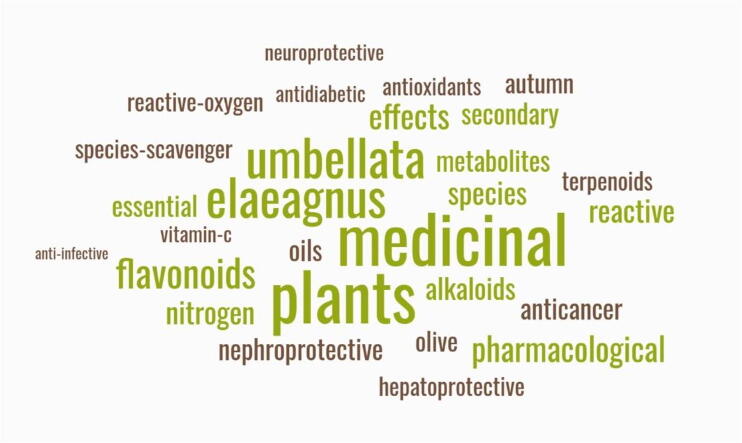

## 1 Introduction

Plants (especially medicinal ones) with history to different civilizations serve the purpose of medicine since ancient times. They were inducted to human diet not only for nutritional value but for use as prophylactics and therapeutic agent in the treatment of different diseases. With a major portion of medicinal plants demarked in the Himalayan belt, in India, it fulfills a major proportion (∼80%) of its demand for use in Ayurvedic medicine, followed by Unani medicine (46%) and a sufficient amount (∼33%) of the allopathic drugs (Samant et al., 2007, Neeta et al., 2011). Though 80% of the drugs are synthesized from plants, their utilization as part of the traditional medical system occurs in the area where they grow (Bauer and Brönstrup, 2014, Pešić and Stanković, 2015). The medicinal value attributed to plants owe to the production of secondary metabolites or bioactive moieties commonly referred to as phytochemicals, which includes a series of flavonoids, alkaloids, terpenoids, essential oils, etc (Tariq et al., 2015, AlSheikh et al., 2020). These phytochemicals unfold important medicinal properties such as analgesic, antioxidant, and others, with a definite physiological action within the complex system of the human body (Zglińska et al., 2021). World Health Organization (WHO) in its report suggested that about 80% of the world’s population still follows the practice of traditional medicine for use in the treatment of different diseases (Sen and Chakraborty, 2017). Lately, a major shift was observed in studying bioactive compounds produced by common dietary items (Fruits, vegetables, herbs, etc) in connection with their isolation followed by identification using advanced methodologies towards the development of potent drug alternatives.

Plants with unique medicinal properties are considered as important source to indigenous system of medicine. In consideration of this, resurgence in following the traditional treatment strategies has led to increase in studies on plants with respect to their wide range of bioactive compounds with strong correlation for use in the treatment of disease or in providing the structural active molecules for use in the development of different drugs (Zglińska et al., 2021). Despite the popularity of plant-based secondary metabolites in protecting plants from different stresses, their use as prophylactics and therapeutic agents in the Indian folk medicine has led to decline of their population in natural habitats that often proceeds with the imposition of restrictions on their use and exploitation as a source of medicine. Herein, we studied the occurence and distribution of an important medicinal plant *Elaeagnus umbellata* in terms of habitat, morphology, traditional importance and importantly production of large number of bioactive compounds in context to their use in the treatment of different human diseases.

## 2 *Elaeagnus umbellata* Thunb.

The Elaeagnus family (Elaeagnaceae; native to Northern Hemisphere) comprises 3 genera with 45–64 species. Of the different members, *Elaeagnus umbellata* Thunb. (also referred to as Autumn Olive) exhibits high medicinal value, with a widespread distribution across the Pir Panjal region of the Himalayas (Dirr, 1990, Ahmad et al., 2005). Its cultivation fulfills the seasonal food shortage for wildlife, besides being used as ornamental, fuel wood, and in making baskets, shelter belts, etc (Munger, 2003). *E. umbellata* is found at 1200–2100 m a.m.s.l and grows at varying temperatures ranging from 43 to 55 °C (Ahmad and Kamal, 2002) and pH range of 5.5–9.5 (Ahmad et al., 2005). It exhibits remarkable differences in traits such as branches size per plant, size and number of thorns on stem, surface area of leaves, number of leaves and fruits per branch, fruit per bunch and their pulp weight, besides showing differences in biochemical profile such as vitamin C, pulp, and measure of seed oil (Sabir et al., 2003). It is planted in arid settings as a protective hedge around farms, homes, and gardens. It exhibits the property to grow at low temperatures and has a tolerance for pruning. It possesses root nodules that attribute it with the property to fix atmospheric nitrogen (Sternberg, 1982, Paschke et al., 1989). It has the ability to prevent soil erosion, besides serving as an attractant to wildlife (Ahmad et al., 2005, Love, 2020). Its ability to withstand high salt concentration and drought conditions makes it a perfect fit to repair sand mounds along the shore and support vegetation along degraded parts of mountainous terrain (Love, 2020, Gardner, 1958, Kim et al., 1993). *E. umbellata* maximize photosynthetic rates despite reduced stomatal conductance and xylem pressure potentials. It continues its photosynthesis even under dry conditions where stomata open early in the day and reach their peak rate before noon throughout the summer (Ahmad et al., 2005, Klich, 2000). *E. umbellata* has an evident benefit in increasing the carbon uptake even under water scarcity conditions, which contributes to its total invasive potential.

## 3 Morphological features of *E. Umbellata*

*E. umbellata* is a deciduous spiny branched shrub of about 2–5 m tall and a diameter of 10 cm with clusters of leaves that are elliptic, oblong, ovate, and alternate (Ahmad et al., 2005, Sather and Eckardt, 1987). It exhibits structural resemblance with plants that grow in shade. For the enhancement of solar capture, the leaves are oriented at horizontal angles (Brantley and Young, 2009). Leaves have smooth margins and blunt tips with sizes of 4–8 cm long and 1–2.5 cm wide. The lower surface is dense, while the upper surface is sparsely white leptitode. Apex is acute or obtuse. Petioles are relatively short dense white leptitode of 0.5–1.0 cm long and densely covered with silvery scales. The leaves have trichomes on both surfaces with the abaxial side being more pubescent (Olson, 2017). Pubescence reduces the leaf absorptance by increasing reflectance (Ahmad et al., 2005, Olson, 2017), thereby lowering the stress induced by the bright light. Representing an adaptive trait to survive in xeric conditions, it protects leaves in a variety of ways during times of stress as is found in the related invasive species *E. angustifolia* (Klich, 2000). Additionally, the presence of trichomes on stem, leaves, etc acts as an adaptive strategy in avoiding high temperature of hot sunny days by maintaining an optimal temperature for optimal photosynthesis (Ahmad et al., 2005, Olson, 2017).

**Fig. 1:**
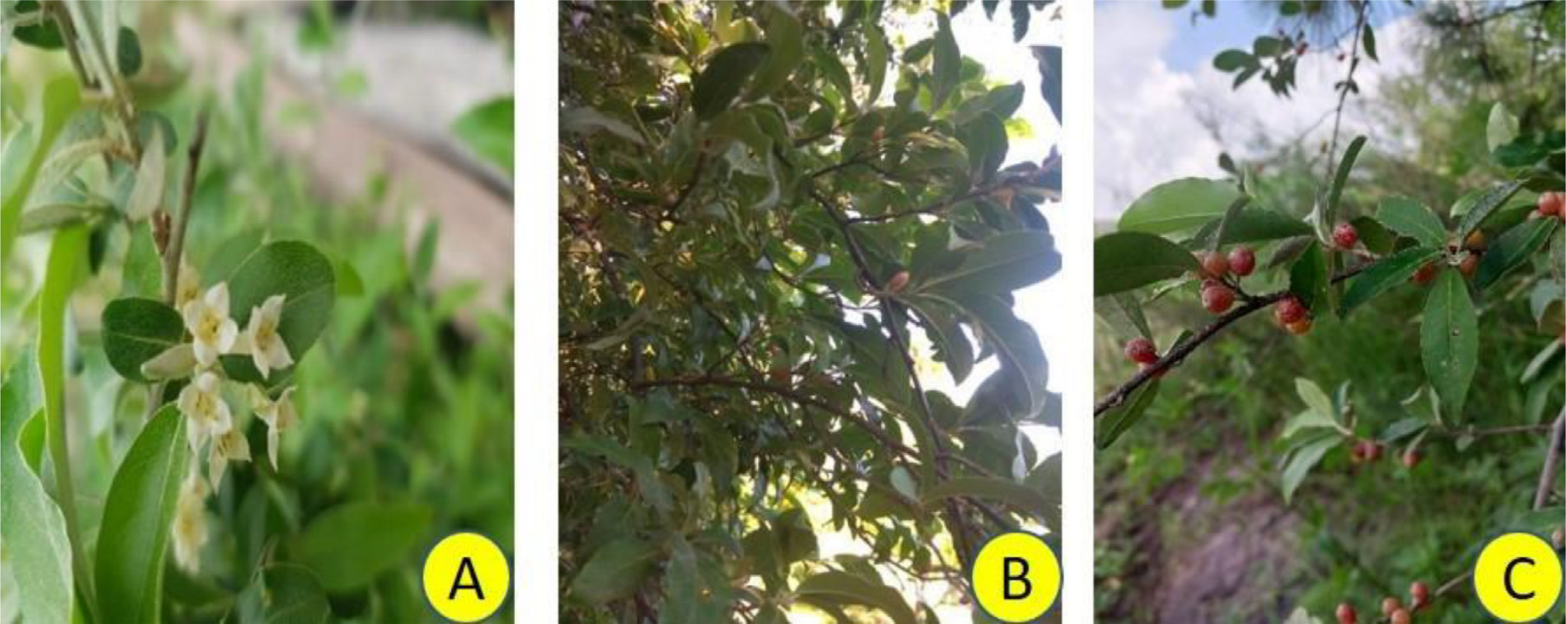
The figure summarizes different stages of the growth of *Elaeagnus umbellata*. A) Flowering, B) Immature, and C) Ripened fruits.

The flowers are cream to white colored with four spreading lobes. The calyx is shorter than its tubular base. The flowers bloom in the spring and with a warm spice fragrance. The fruits referred to as pseudodropes are spherical in shape with a length of 7 mm. Acting as an attractant, fruits are silver-white (at the young stage) in color that changes to crimson red once it matures (Sather and Eckardt, 1987) (Fig. 1). The fruits have the ability to retain their properties even after 15 days of storage at room temperature (Parmar and Kaushal, 1982, Kim et al., 2016). The pigment responsible for red color of the fruit is lycopene (Fordham et al., 2001). The fruits abundant on growing branches are delicious with a sweet to acidic flavor. The fruits are eaten raw or processed into sauces, and fruit rolls, or used as a tomato substitute. The fruits rich in lycopene are effective in exerting protection against chronic diseases such as myocardial infarction (Kohlmeier et al., 1997, Patel, 2015) and prostate and other types of cancers (Giovannucci et al., 1995, Clinton, 1998). The plant is used as an adornment because of its lovely blooms and silvery foliage leaves.

## 4 Phytochemistry of *E. umbellata*

**Fig. 2:**
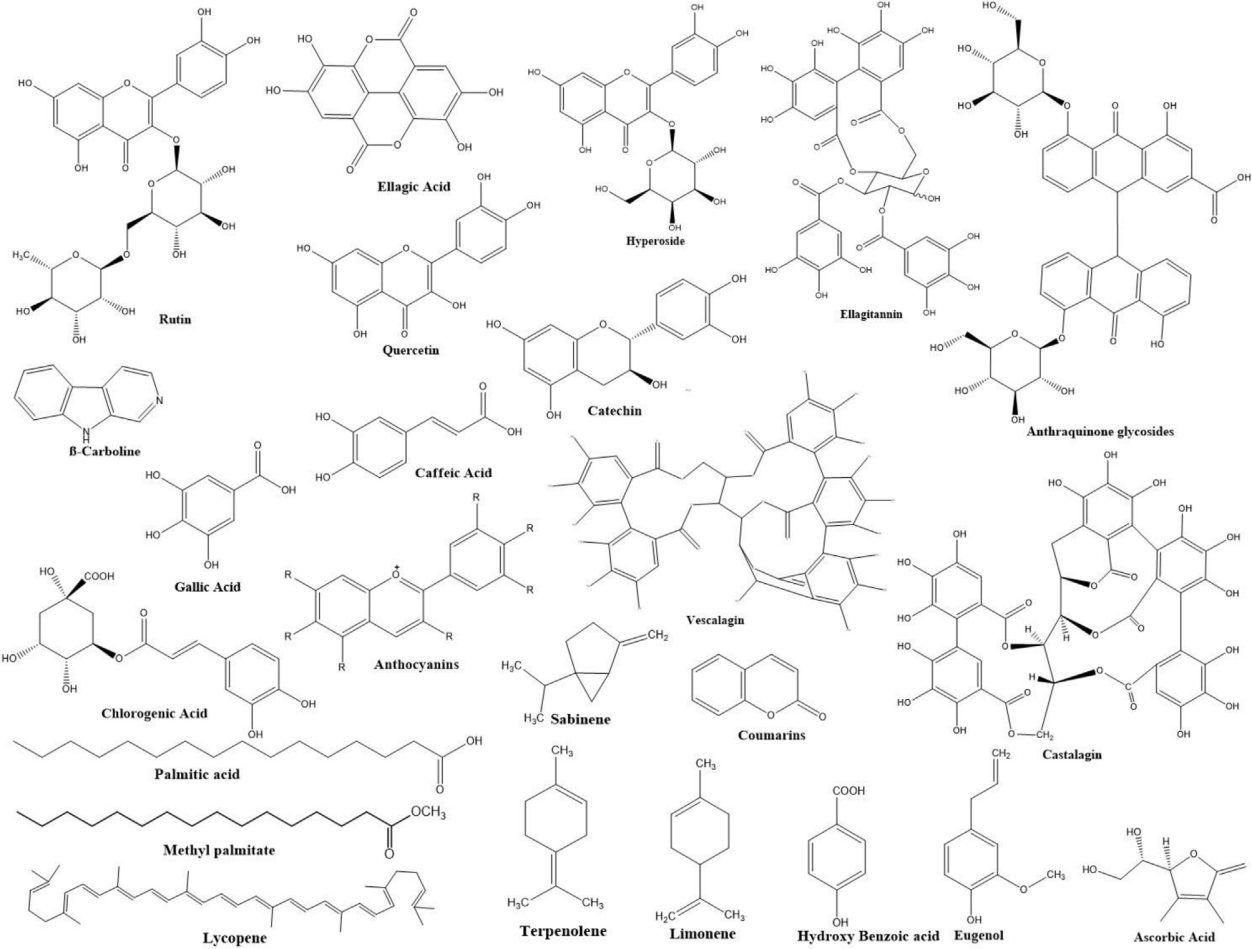
Structures of bioactive compounds reported in *Elaeagnus umbellata.*

Phytochemicals, in principle the secondary metabolites produced by plants, exert health-promoting benefit that helps in preventing the occurrence of chronic diseases. They are gaining relevance as health-promoting agents that extends numerous benefits in humans following a phytochemical-rich diet (He et al., 2007, Kavanaugh et al., 2007, Christen et al., 2008). The phytochemicals produced by *E. umbellata* include phenolics, flavonoids, carotenoids, tannins, alkaloids, and saponins, besides being a source of essential minerals and vitamins particularly vitamin C (Fig. 2). Regular consumption of nutrient-rich diet helps in preventing production of free radicals and as such in reducing or delaying oxidative stress (Liu, 2004). Nearly, 84 bioactive constituents have been isolated from *E. umbellata* of which eugenol, 4-methoxy anisole, 2-nonenal, palmitic acid, 3-hexenylacetate, fatty acid methyl ester, phenylacetaldehyde, 4-methyl phenol, 2-hexanal, and methyl palmitate are the major ones observed in the extract of floral volatiles (Potter, 1995). It also serves as source of heterocyclic alkaloids having indole skelton and have great importance as anticancer, antihypertensive, antiarrhythmic, antimalarial, and sedative for use in biological and therapeutic purposes (Gamba et al., 2020, Massagetov, 1946).

The fruits of *E. umbellata* is promoted as beneficial ingredient in Western Chinese, Koreans, and Japanese diet owing to its ability in being effective against cancer, hepatitis and liver disorders, fractures, injuries, and diarrhoea (Gamba et al., 2020, Pinto et al., 2013). In a study, a series of seven novel tannins (elaeagnatins A-G) were observed in the leaf extract along with fifteen other known tannins (Ito et al., 1999, Ito et al., 1999). While studying autumn olives, Perkins-Veazie et al. (Perkins-Veazie et al., 2005) found them to have a high phenolic content and suggested correlation of the biological activity of phytochemicals with their concentration and structure attributes. Screening of the different *E. umbellata* genotypes revealed them to have a high phenolic (168.9 to 258.1 mg/100 g) and carotenoid (43.4 to 59.3 mg/100 g) content (Wang and Fordham, 2007). *E. umbellata* being rich in saponins are known to reduce the risk for cancer development, besides offering a regulatory check of lipids, and blood glucose levels. A diet high in saponins can help prevent dental cavities and platelet aggregation, as well as act as a lead poisoning antidote (Shi et al., 2004). Supplementation of alcoholic extract of *E. umbellata* with magnesium oxide was found effective in the treatment of athlete’s foot disease (Potter, 1995). Limonene - the most represented compound present in *E. umbellata*; is used to treat breast cancer (Liu, 2004)*.*

The berries of *E. umbellata* being rich in phenolics, anthocyanins, flavonoids, and tannins exert beneficial effects through reduction in the macromolecular oxidation via, decrease in the oxidative stress (Veberic et al., 2015). The quantity of organic acid such as citric, malic, and oxalic acid varies significantly during different stages of ripening and was found dependent on the climatic conditions, soil, and cultivar choice (Gamba et al., 2020). Exerting a strong antioxidative effect, anthocyanins effectively help in reducing the occurrence of diseases including cancer, diabetes, etc (Yousuf et al., 2016). The berries of *E. umbellata* also have a rich content of carotenoids such as lycopene, lutein, β-carotene, etc. Lycopene from *E. umbellata* has been shown to be 17 times more abundant than in fresh tomatoes and may be responsible for the treatment of cancer and myocardial infarction (Fordham et al., 2001, Kohlmeier et al., 1997, Patel, 2015, Giovannucci et al., 1995). The berries are rich in proteins, pectin, carbohydrates, and acids, besides being a good source of vitamin A, C, and E (Gamba et al., 2020). *E. umbellata* fruits (100 g) contain moisture content of 64.9 g, soluble solids (14.5 g), acids (1.51 g), total sugar (8.34 g; with reducing (8.13 g) & non-reducing (0.23 g)), and a high content of vitamin C (12.04 mg) (Ahmad et al., 2005). It is also a rich source of phosphorus (0.054), calcium (0.049), magnesium (0.033), potassium (0.346), and iron (0.007) (Ahmad et al., 2005, Gamba et al., 2020). The fruits of this plant are used to treat diarrhoea, itch, and foulness, besides being used in making fruit rolls, juices, and condiments (Pei et al., 2015). The berries can be used raw or cooked. The flowers of *E. umbellata* are not showy and exhibit a strong characteristic odor which is due to phenolics (Pei et al., 2015). Having a high content of sugar, and fatty acids, leaves of *E. umbellata* are used to treat bowel disorders in China and Japan.

## 5 Pharmacological properties of *E. Umbellata*

*E. umbellata* shows production of different classes of secondary metabolites, prominent being alkaloids, polyphenols, terpenes, and others (Uddin and Rauf, 2012). Studies on the pharmacological front have revealed them to have properties of treating hepatic dysfunctioning and in exerting potent antibacterial, antiproliferative, antioxidant, and phytotoxic effects (Khattak, 2012, Rafique et al., 2016, Wang et al., 2019, Nazir et al., 2020, Nazir et al., 2018, Zulfiqar et al., 2022). The pharmacological properties of the plant are summarized in Table 1.

**Table 1:** Table summarizes information of the bioactive compounds, their origin and application in the treatment of different diseases.

**Bioactive compound**	**Class**	**Parts used**	**Medicinal uses**	**References**
Ellagic acid	Benzoic acid	Berries	Inhibits cancer cell progression and improves efficacy of drugs against cancer	(Gamba et al., 2020, La Vignera et al., 2022)
Gallic acid	Benzoic acid	Berries	Anti-oxidant, anti-inflammatory and anti-neoplastic activity	(Gamba et al., 2020)
Ascorbic acid	Vitamin C	Berries	It helps to heal wounds and enhance the absorption of iron from plant foods. It also supports the immune system.	(Gamba et al., 2020, Zulfiqar et al., 2022)
Terpenolene	Monoterpenes	Berries	It is used to treat anxiety and insomnia	(Gamba et al., 2020)
Chlorogenic acid	Cinnamic acids	Berries	It reduces the inflammation and modulate inflammatory and neuropathic pain in animal models.	(Gamba et al., 2020)
Limonene	Monoterpenes	Berries	It has anticancer, anti-oxidant, antiviral, anti-inflammatory, and gastroprotective	(Gamba et al., 2020)
Sabinene	Monoterpenes	Berries	It has anti-fungal and anti-inflammatory properties	(Gamba et al., 2020, Cao et al., 2018)
Caffeic acid	Cinnamic acids	Berries	It has the anticancer, anti-oxidant and anti-inflammatory potential	(Gamba et al., 2020, Ishaq et al., 2015)
Rutin	Flavanols	Berries	It is commonly used for autism, aging skin, airways infections caused by exercise. It has also the anti-oxidant and anti-inflammatory properties	(Gamba et al., 2020, Nazir et al., 2021)
Quercetin	Flavanols	Berries	It helps to protect against heart disease and cancer	(Zglińska et al., 2021, Gamba et al., 2020)
Castalagin	Catechins	Berries	It promotes bacterial cell wall disruption via, modulation in the assembly of peptidoglycans	(Gamba et al., 2020, Araújo et al., 2021)
Catechin	Catechins	Berries	It is used in the treatment of chronic diseases in humans, such as inflammatory bowel disease (IBD). It has also anticancer, antioxidant, antimicrobial, antidiabetic, and anti-inflammatory properties.	(Gamba et al., 2020, Azeem et al., 2022)
Hydroxybenzoic acid	Benzoic acid	Berries	It possesses anti-inflammatory, antimutagenic, antioxidant, hypoglycemic and antimicrobial properties	(Gamba et al., 2020, Xu et al., 2021, Kalinowska et al., 2021)
β-carboline	Alkaloid	Bark	It has sedative, anxiolytic, hypnotic, anticonvulsant, anticancer, and antimicrobial, activities.	(Paudel et al., 2020, Tolkachev et al., 2008)
Coumarins	Alkaloid	Whole plant	It has anti-inflammatory, anticancer, anticoagulant, antihypertensive, antimicrobial, anti-tuberculous, anticonvulsant, antiadipogenic, antihyperglycemic, and neuroprotective activities	(Paudel et al., 2020, Sharifi-Rad et al., 2021, 2021., 2021.; Minhas et al., 2013)
Anthraquinone glycosides	Glycoside	Aerial parts	It is antifungal in nature. It promotes normal kidney function and modulates inflammation via, inhibition of the enzyme cyclooxygenase	(Paudel et al., 2020, George et al., 2014, Rawat et al., 2002)
Ellagitannins	Tannins	Leaves	It has anti-inflammatory, anticancer, antioxidant, prebiotic, and cardioprotective properties	(Ozen et al., 2018, Ito et al., 1999, Ito et al., 1999)
Eugenol	Allylbenzene	Flower	It is used to treat toothache and more rarely be taken orally to treat gastrointestinal and respiratory complaints	(Gamba et al., 2020, Nazir et al., 2020, Nazir et al., 2018, Nazir et al., 2021)
Palmitic acid	Fatty acid	Flower	It acts as anti-inflammatory, promotes metabolic activities, and supports skin health	(Zglińska et al., 2021)
Methyl palmitate	Fatty acid methyl ester	Flower	It prevents development of fibrosis via, inhibition of NF-κB in rats	(Zglińska et al., 2021)
Lycopene	Carotenoid	Berries	It is a powerful antioxidant, antidiabetic, and possess neuroprotective and anticancer properties	(Gamba et al., 2020, Kaur et al., 2011)
Anthocyanins	Flavonoids	Berries	Reduce risk of cancers, beneficial to improve brain functions, protect from UV rays and also have antimicrobial activity	(Gamba et al., 2020, Ozen et al., 2018)
Kaempferol-O-hexidose	Polyphenol	Berries	It possesses strong anti-inflammatory and anticancer properties	(Spínola et al., 2019)
Vescalagin	Tannins	Berries	It promotes bacterial cell wall disruption via, modulation in the assembly of peptidoglycans	(Gamba et al., 2020, Araújo et al., 2021)
Hyperoside	Cinnamic acids	Berries	It is commonly used as an anti-inflammatory. It also has antioxidant and antimicrobial properties.	(Gamba et al., 2020, Mahomoodally et al., 2018)

### 5.1 Antioxidant activity

**Fig. 3:**
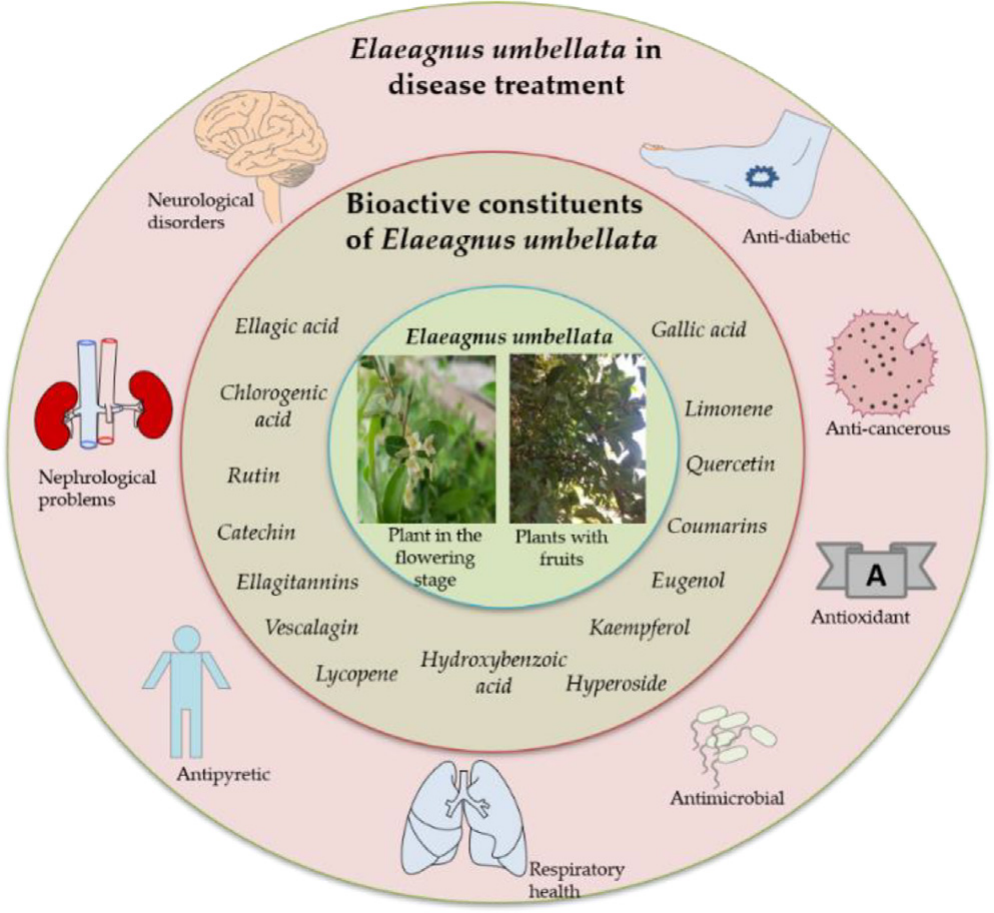
Figure depicting the potential of a wide range of bioactive compounds of *Elaeagnus umbellata* in exerting benefits towards the treatment of different diseases.

Most diseases in humans have direct or indirect involvement of free radicals. The free radicals are kept in check by antioxidants that regulate their production within the system (Jan et al., 2011). *E. umbellata* is an excellent source of nutrients and antioxidants, and as such attribute beneficial effects to the health of an individual. In *E. umbellata*, a diverse variety of antioxidants found in fruits (berries) exerts synergistic effects in countering free radicals and avoiding damage caused by oxidative stress (Wang et al., 2019, Zulfiqar et al., 2022, Steinberg, 1991). The methanolic extract of berries was found to exert an antioxidant effect in a dose-dependent i.e., a dose of 20, 40, 60, 80, 100, and 120 μg/ml showed scavenging activity of 10.7, 26.7, 49.0, 69.3, 84.9 and 90.2%, respectively (Ahmad et al., 2005). Though acetone and aqueous extracts showed a similar pattern of scavenging activity, however, it was found that the methanolic extract exhibited a lower EC_50_ value (97.3 μg/ml) compared with the acetone extract showing an EC_50_ value of 188.0 μg/ml (Khanzadi, 2012). Zglinska et al. (Zglińska et al., 2022) explore the effect of methanol-acetone extract from fruits on the antioxidant capacity of healthy human cells that had undergone oxidative stress. They found the extract effective in boosting cell viability rather than exhibiting any toxicity. On examining its effect on fibroblasts, the extract was found effective in decreasing the expression of chemokine and as such H_2_O_2_-induced cell death (Iannuzzi et al., 2020). On studying the effect of essential oils through ABTS and DPPH assays, it was found that a concentration of 1000 µg/ml with IC_50_ values of 105 and 70 µg/ml shows a free radical scavenging capacity of 88.30 ± 0.81 and 85.24 ± 0.63 against citric acid used as a positive control (Nazir et al., 2021). Of the different extracted essential oils; Octadecanoic acid exhibits a strong antioxidant potential (Wang et al., 2007). The others in the category of essential oils such as linoleic acid, phytol, p-vinyl guaiacol, stearic acid, decanoic acid, etc, displayed reasonable antioxidant activity (Khanzadi, 2012, Cai et al., 2004). Regardless of the reagent used, the lyophilized berries were distinguished by higher antioxidant activity than dried fruit (Zglińska et al., 2022). Despite the fact that aqueous extract exhibits better antioxidant impact in the liver and brain of mice (Ishaq et al., 2015), antioxidant status and FRAP test of the dried berries revealed higher activity for methanolic extract than aqueous one (Zglińska et al., 2022, Ozen et al., 2017). Zulfiqar et al. (Zulfiqar et al., 2022) reported that silver nanoparticles synthesized for fruit extract showed to have an antioxidant potential of 43.38 µg/ml (IC_50_ value) in DPPH assay and an inhibition of 69%. Compared with fruits, leaves having a good proportion of acids (fumaric, 4-hydroxybenzoic acid, etc), and flavonols (rutin, neohesperdin, and hesperdin) also exhibit strong antioxidant properties (Zglińska et al., 2021, Zglińska et al., 2022). In the screen of antioxidant potential in DPPH and ABTS assay, IC_50_ values of 40, 45, and 60 g/ml and 57, 70, and 120 g/ml were observed for the leaf extract prepared in chloroform, ethyl acetate, and butanol (Nazir et al., 2021) (Fig. 3).

### 5.2 Anticancer activity

Cancer is the second largest cause of mortality worldwide. Its development starts in a multistage process from a normal cell to a precancerous lesion and finally to a malignant tumor (Chan et al., 2019, 2019.). In 2018, it was estimated that around 9.6 million people died from cancer alone (Chan et al., 2019, 2019.). As phytonutrients are considered beneficial in preventing the incidences of diseases such as heart disease, cancer, and others, an increased intake of fruits and vegetables was observed among the diet of consumers (WCRF, AICR., 2007). Of them, antioxidants from plant sources are well recognized as effective in avoiding arteriosclerosis, cancers, cardiovascular diseases, cellular injuries, inflammations, neurological problems, and dysregulation of the immune system (Steinberg, 1991, Ozen et al., 2017). Besides having a good percentage of lutein, α-carotene, and other similar compounds, Fordham et al. (Fordham et al., 2001) reported that *E. umbellata* fruits contain 7–17 times more lycopene than tomatoes with its role in extending protection against a wide range of malignancies. Ellagic acid extracted from the fruits of *E. umbellata* prevents cancer cell growth and as such improves the efficacy of anticancer drugs (La Vignera et al., 2022). Catechin belonging to the class catechins was extracted from the berries of the plant which shows anticancer properties (Azeem et al., 2022). It was studied that Anthraquinone glycosides (a glycoside extracted from the whole plant) inhibits excessive renal tubular cell proliferation (Paudel et al., 2020, Rawat et al., 2002). In another study, it was shown that Caffeic acid extracted from the berries exhibit higher anticancer potential (Gamba et al., 2020, Kim et al., 2005).

### 5.3 Anticholinesterase activity

Alzheimer’s disease (a neurodegenerative disorder) is characterized by an increase in oxidative stress, β-amyloid build-up, low levels of the neurotransmitter acetylcholine (ACh), and a decrease in cholinergic transmission (Perry et al., 1998, Vickers et al., 2016, Niranjan, 2018). According to the World Alzheimer's Organization's 2015 estimate, there are approximately 46.85 million people worldwide who are affected by Alzheimer's disease and other kinds of dementia (Kuca et al., 2016, Kumar et al., 2018). They also anticipate that by 2030, this number will have doubled, and by 2050, it will be three times than its current level. The decrease in cholinergic transmission occurs due to the breakdown of the neurotransmitter acetylcholine (ACh) by acetylcholinesterase (AChe) and butyrylcholinesterase (BChe). In this process, inhibition of the AChe and BChe enzymes helps in maintaining the Ach levels, thereby relieving the body of symptoms pertaining to dementia, Parkinson, and Alzheimer diseases (Rahman and Choudhary, 2001, Mangialasche et al., 2010). The secondary metabolites such as alkaloids, found in the *E. umbellata* extracts exhibit considerable cholinesterase activity; thereby serving as a source of cholinesterase inhibitors (Mehta et al., 2012, Murray et al., 2013). Phyto-constituents of the *E. umbellata* fruits including different acids (chlorogenic, ellagic acid, and gallic acid) and phloroglucinol were found attributing anti-cholinesterase activity both under *in vitro* and *in vivo* conditions (Mehta et al., 2012, Murray et al., 2013, Ghias and Rauf, 2012). Treatment of animal models with ellagic and chlorogenic acids showed positive effects in terms of reducing oxidative stress and a subsequent enhancement of the cognitive effects that attribute neuroprotection against scopolamine-induced amnesia (Kwon et al., 2010, Jha et al., 2018). The chloroform extract helps in restoring the cholinergic activities via, inhibition of the acetylcholinesterase enzyme, leading to the retention of higher levels of acetylcholine levels in the brain which is considered crucial for cognitive impairment. Similarly, Nazir et al. (Nazir et al., 2021) found that essential oils of *E. umbellata* effectively inhibit the enzymes AChe and BChe, thus claiming that essential oils of *E. umbellata* hold potential for use as a medication in the treatment of neurological diseases. The well-known alkaloids Galantamine and rivastigmine obtained from *E. umbellata* are used to treat Alzheimer's disease (Ahmad et al., 2005, Murray et al., 2013). Galantamine, donepezil, and rivastigmine are the only FDA-approved acetylcholinesterase inhibitors for Alzheimer's disease (Mangialasche et al., 2010). As antioxidant therapy has shown positive effects in improving cognitive impairment and behavioral patterns, supplementation of natural products rich in antioxidants is believed to be effective in reducing the chances of neuronal cell death and as such delaying the early onset of the disease (Erejuwa et al., 2012, Vina et al., 2011).

### 5.4 Anti-inflammatory activity

The fruit extract of *E. umbellata* exhibits anti-inflammatory property owing to its ability in controlling inflammation; thereby increase in its usage as a functional additive (Zglińska et al., 2021). The extract was found effective in increasing the production of metalloproteinases such as TIMP-1 (Tissue inhibitor of metalloproteinases-1), and MMP-9 (Matrix metalloproteinase-9), suggesting that collagen may be guarded against breakdown under oxidative stress. These traits, along with increase in the expression of brain-derived neurotrophic factor (BDNF) highlight the possibility of its use in the management of neuro-inflammatory conditions concerned to Alzheimer's and Parkinson's diseases (Zglińska et al., 2021). While studying LPS-stimulated cells, Kang et al. (Kang et al., 2020) observed that gallic acid from the leaves of *E. umbellata* is capable of inhibiting nitric oxide production, thereby exhibiting a strong anti-inflammatory activity. Kaempferol present in the leaves of the *E. umbellata* shows strong anti-inflammatory activity and inhibits the T- cell proliferation at the concentration of 100 µM (Kang et al., 2020, Wang et al., 2018). Phytoene extracted from the *E. umbellata* is also found to have anti-inflammatory properties (Paudel et al., 2020). Another component adiponectin has been found effective in reducing the level of pro-inflammatory cytokines, thus functioning as an important anti-inflammatory factor (Polyzos et al., 2010). In another study methyl palmitate extracted from the flower of the plant reduce the chances of fibrosis in rats following treatment with CCl4 and bleomycin via, inhibition of the NF-κB (Zglińska et al., 2021).

### 5.5 Anti-diabetic activity

Diabetes (mostly Type 2) caused by less production of insulin and insulin resistance show 2–10 times higher mortality risk owing to vascular (both macro and micro) diseases (Manson et al., 1991, King et al., 1998, Cheng, 2005). As α-amylase causes the breakdown of starch to disaccharides, it is acted upon by α-glucosidase that hydrolyzes disaccharides into simple sugars like glucose for absorption at the intestinal surface; thereby resulting in postprandial hyperglycemia (Dhital et al., 2013). Diabetes representing a postprandial hyperglycemic condition is often associated with increase in the oxidative stress (Brownlee, 2001) and risk for the development of cardiovascular complications (Bonora and Muggeo, 2001, Ceriello, 2005). Postprandial hyperglycemia is controlled by inhibiting the enzyme α-glucosidase that impairs digestion of the dietary carbohydrates in the intestines (Standl et al., 1999). Affecting the absorption of monosaccharides, inhibition of α-glucosidase flattens the postprandial increase of the blood glucose; thereby results in the alleviation of postprandial hyperglycemia condition (Miura et al., 1998, Heo et al., 2009). Acarbose - a potent hypoglycemic agent, lowers the incidences of hyperglycemia and hyperinsuliminea via, improved insulin sensitivity that proceeds with full control over hyperglycemia (Standl et al., 1999, Breuer, 2003). Despite bringing benefits of controlling the hyperglycemic condition, oral intake of acarbose was found to cause gastrointestinal problems such as abdominal discomfort, flatulence, and diarrhoea (Bressler and Johnson, 1992).

Diabetes serve as an important risk factor in the development of cardiovascular complications. In such a scenario, plant polyphenolics such as flavonoids, etc are often correlated positively with inhibition of the α-glucosidase activity (Miura et al., 1998, Heo et al., 2009). As the antidiabetic effect includes a regulatory check of glucose absorption, reduction in inflammation, protein glycation, and oxidative stress, consumption of *E. umbellata* (often referred to as Autumn olive berries, AOB) fruits is considered a safe and cost-effective approach in reducing the hyperglycemic condition (Nazir et al., 2018, Harris et al., 2014, Edirisinghe and Burton-Freeman, 2016). Consumption of AOB was found effective in inhibiting α-glucosidase, besides having a significant effect on the expression of adiponectin that plays important role in sensitizing insulin action (Nigro et al., 2014). As diabetes often increases inflammation and as such diabetic associated complications (Alexandraki et al., 2008), adiponectin was found effective in reducing the levels of pro-inflammatory cytokines and as such is capable of exerting an anti-inflammatory effect (Polyzos et al., 2010). Together, AOB was found effective to overcome insulin sensitivity via, inhibition of the α-glucosidase and a subsequent increase in the expression of hormone, adiponectin (Nigro et al., 2014). AOB serves as a potent hypoglycemic agent in flattening the glucose levels towards achieving the goal of preventing the occurrence of diabetes and diabetes-associated complications (Kim et al., 2019).

The essential oil of fruits in *E. umbellata* also exhibits antidiabetic potential (Nazir et al., 2021). α-linolenic acid reported in the fruit essential oil through GC–MS was found to have antidiabetic activities (Dhital et al., 2013, Jovanovski et al., 2017). Phytol (3, 7, 11, 15-tetramethyl-2-hexadecen-1-ol) also shows strong antidiabetic activity (Elmazar et al., 2013). Similarly, other components of the essential oils of *E. umbellata* like stearic acid, humulene epoxide, and ascorbic acid were reported to possess antidiabetic potential (Zhao et al., 2017, Lalitha et al., 2015). Proanthocyanidins present in berries improve insulin sensitivity via, enhancement in adiponectin expression in rats fed with a high fructose diet (Meeprom et al., 2011). Lycopene in berries exhibits strong antioxidant potential; thereby is effective against the development of oxidative stress (Veazie et al., 2005). The methanolic, chloroform, and ethylacetate extract of berries exhibits a strong inhibitory effect against different enzymes such as α-amylase, α-glucosidase, etc (Wang et al., 2000). The antihyperglycemic effect of the extract also includes reduction in triglycerides (TGS), low-density lipoproteins (LDL), and cholesterol level. Additionally, the extract was found to exhibit an anti-hepatoprotective effect among the diabetic control group demarked by reduction SGOT, SGPT, and ALP levels.

### 5.6 Antimicrobial activity

Berries and leaves of *E. umbellata* contain a high amount of phenolic compounds such as hydroxybenzoic acids, besides having a good proportion of flavanols that attribute it with strong antibacterial activity (Chopra et al., 1986,, Gairola et al., 2021). The seeds contain a good percentage of acids such as chlorogenic acid, gallic acid, etc. Their presence attributes *E. umbellata* with the property of being effective in the treatment of bacterial infections. The essential oils extracted from *E. umbellata* are effective in destroying the cell membrane and cell wall, besides causing impairment in the membrane permeability that causes release of their intracellular contents, disturbance of the nutrient uptake and electron transport system, and overall hindrance with the membrane function (Minhas et al., 2013). The antimicrobial activity of *E. umbellata* extracts (chloroform, *n*-hexane, ethyl acetate, and methanol) against varied pathogenic strains (*S. epidermis, S. aureus*, *B. subtilis*, *E. coli,* and *K. pneumonia*)*,* shows a different level of inhibition. Gram-positive bacteria were shown to be resistant to all fractions, whereas gram-negative bacteria were completely inert. Among all the fractions ethyl acetate were exhibiting the highest activity against *S. epidermis.* The fraction of ethyl acetate and methanol also showed significant effectiveness against *S. epidermis, S. aureus,* and other bacterial strains (Ghias and Rauf, 2012). Similarly, the activity of the extract prepared from the flower in ether was found effective against broad range of pathogenic strains (*B. subtilis, E. coli, S. aureus* and others) (Minhas et al., 2013, Mubasher et al., 2007). The ethanolic extract of leaves shows strong activity against strains belonging to both gram +ve and gram -ve bacteria, while the aqueous extract of berries against *B. subtilis* showed a relatively small zone of inhibition but strongly inhibits the growth of *S. aureus* and *E. coli* (Minhas et al., 2013, Mubasher et al., 2007)*.* The aqueous extract of berries was shown to have no effect on multi-drug-resistant *P. aeruginosa*, while growth was inhibited by acetone extract (Nazir et al., 2021).

## 6 Conclusion and future perspectives

Plants comprising a large proportion of the human diet fulfill the daily caloric requirements (both organic and inorganic) needed to perform biological activities on a routine basis. With varied composition and nutritional value, they contribute significantly as a source of proteins, vitamins, minerals, etc, that defend the body from chronic illnesses and varied human diseases. Of the different plants, *E. umbellata* rich in bioactive constituents (flavonoids, phenolics, etc), vitamins (A, C & E), and minerals (zinc, iron, calcium, and others), is considered indispensable to the life of different organisms including humans. Besides acting as a source of energy, it fulfills the nutritional requirements for maintaining normal health by having active participation in the growth, maintenance, and routine functions of the body. In the recent past, plant-based phytochemicals are gaining popularity for use in the treatment of different diseases. The bioactive constituents of *E. umbellata* have been well-researched for their use in the treatment of different diseases, besides having considerable antioxidant, anticancer, anti-inflammatory, anticholinesterase, and antimicrobial potential. It not only helps in controlling blood glucose levels via, inhibition of α-glucosidase and alteration in the expression of hormone, adiponectin, but also plays an active role in the amelioration of cognitive dysfunction and in the restoration of acetylcholine levels. In short, the consumption of a phytochemical-rich diet fulfilling the daily requirements ascertains how significant these nutrients are to the health and well-being of humans.

In the current scenario of an increase in the emergence of drug resistance that has raised serious concerns pertaining to human health, there is an urgent need to strategize the policies regarding the exploration of plant species as a possible therapeutic option. With less production of new drugs and increased cases of drug resistance, there is an urgent need for possible exploration of medicinal plants as a possible source of drugs to combat the menace of drug resistance. Considering the economic burden and substantial health concerns, medicinal plants exhibiting potent drug properties need exploration for new bioactive moieties and their synthesis pathways with a possible trigger towards manipulation for enhancing their production and perpetuation as a possible drug molecule.

## 7 Methods adopted for search of the literature

The bibliometric research for the article was carried out on a broader range; starting with medicinal plants of the family Elaeagnaceae, the health benefits of *Elaeagnus umbellata*, its role in different diseases (1950–2022) across different databases (PubMed, Scopus, Web of Sciences, and others). The data collected was differently classified and explored based on origin, role in traditional medicine, structural properties, bioactive compounds, and their pharmacological properties.



## Author contributions

All authors contributed equally to the writing of the contents present under different sub-headings of the manuscript. All authors have read and agreed to the published version of the manuscript.

## Funding

Author Arif Tasleem Jan would like to thank DST-SERB (CRG/2019/004106) and J&K Science Technology and Innovation Council (JK ST&IC/SRE/996–998), India for support in terms of the grant.

## Declaration of Competing Interest

The authors declare that they have no known competing financial interests or personal relationships that could have appeared to influence the work reported in this paper.
